# Efficacy of Weikang Pian in Patients with Functional Dyspepsia: A Double-Blind, Randomized, Placebo-Controlled Clinical Trial

**DOI:** 10.1155/2019/4827046

**Published:** 2019-11-06

**Authors:** Lijing Yan, Lijin Yu, Linlin Zhao, Dongsheng Wang, Dilan Qin, Haiwei Fan, Ling Cheng, Musen Qiu, Xiao Chen, Lu Zhou, Juan Qiu, Jiamei Yao, Wenbo Wang, Xinjian Qiu

**Affiliations:** ^1^Institute of Integrated Traditional Chinese and Western Medicine, Laboratory of Ethnopharmacology, Xiangya Hospital, Central South University, Changsha, Hunan 410008, China; ^2^Department of Health Management, The Third Xiangya Hospital of Central South University, Changsha, Hunan 410013, China; ^3^Hunan Province Environmental Monitoring Centre, State Environmental Protection Key Laboratory of Monitoring for Heavy Metal Pollutants, Changsha, Hunan 410019, China; ^4^State Key Laboratory of Innovative Natural Medicine and TCM Injections, Ganzhou, Jiangxi 341000, China; ^5^Department of Nuclear Medicine, The Third Xiangya Hospital of Central South University, Changsha, Hunan 410008, China; ^6^National Clinical Research Center for Geriatric Disorders, Department of International Medical, Xiangya Hospital, Central South University, Changsha 410008, Hunan, China

## Abstract

**Background:**

FD (functional dyspepsia) is a common functional gastrointestinal disorder, which lacks effective and safe treatment. Chinese herbal medicine (CHM) has been applied in FD treatment for thousands of years with satisfactory clinical outcomes. Zhishi is a classical traditional Chinese medicine used to treat FD. Weikang pian (WKP) is made of flavonoids extracted from zhishi which could effectively alleviate the symptoms of FD. This research aimed to assess the efficacy and safety of WKP in FD treatment.

**Methods:**

This was a randomized, double-blinded and placebo-controlled clinical trial. The patients were diagnosed as FD according to RomeIII criteria. Then, FD patients were selected and assigned randomly to either WKP or placebo group. The subjects randomly received WKP or placebo for 4 weeks with 4 tablets each time, 3 times daily. The single dyspepsia symptom (SDS) scale and the gastric emptying function were measured before and after the treatment. Moreover, the safety of the trial and patient compliance were evaluated.

**Results:**

A total of 60 FD patients were eventually enrolled in the trial, among them 45 patients in the WKP group and 15 patients in the placebo group. The primary outcome was the SDS scale, including assessments of postprandial distension, early satiety, epigastric burning, and pain. The secondary outcome was the gastric emptying function. Compared with the placebo group, the symptoms of FD in the WKP group were relieved after 4 weeks of treatment (*P* < 0.05). Some minor changes appeared in the four groups, but there were no significant differences in gastric emptying parameters of GER (2-hour gastric emptying rate) and GET/2 (gastric semiempty time) (*P* > 0.05). Severe adverse events were absent. The compliance to treatment was 94%–96%, and there was no significant difference between the groups.

**Conclusion:**

WKP can relieve FD symptoms to some extent. This trial is registered with Chinese Clinical Trial Registry (ChiCTR): CTR 20132482.

## 1. Background

Functional dyspepsia (FD) is featured by recurrent or chronic fullness in upper abdomen, belching, early satiety, bloating, epigastric algesia, vomiting, nausea, regurgitation, and loss of appetite and burning [1]. Studies have shown that 40% of the population suffered from FD [2]. Besides, FD seriously threatens human health and quality of life and presents a substantial socioeconomic burden. [3, 4] There are still no satisfactory therapies for FD, and the standard treatment has not been established yet [5]. Therefore, effective and safe therapies are urgently needed [6].

Complementary and alternative medicine is composed of traditional Chinese medicine (TCM), traditional Persian medicine (TPM), etc. CHM has been applied in FD treatment for thousands of years in China with satisfactory clinical outcomes. In addition, some exciting evidence about the possible efficacy of CHM in treating FD has been reported [7–12]. Therefore, complementary and alternative medicine should be taken seriously in FD treatment. Zhishi (*Citrus aurantium* L., Aurantii Fructus Immaturus, Rutaceae family) is a classical TCM for FD treatment [13]. WKP is made of flavonoids extracted from zhishi which could effectively and safely alleviate the symptoms of FD. In addition, previous studies have shown that Aurantii fructus immaturus flavonoid (AFIF), the major effective constituent of AFI, could promote gastric emptying process in FD rats [14].

This study aims to evaluate the effects of WKP in FD treatment and provide evidence for application of WKP to FD patients.

## 2. Methods

### 2.1. Ethical Approval and Registration

This was a double-blinded, randomized and placebo-controlled clinical trial, which was designed to assess outcomes and safety of WKP in treating FD. Approval of drug research was obtained from the State Food and Drug Administration. It was conducted in accordance with Clinical Guideline of New Drug for Traditional Chinese Medicine, the Helsinki Declaration and Guidelines for Good Clinical Practice, with approval from China SFDA (No: 2008L04094). This trial was approved by the Ethics Committee of Xiangya Hospital of Central South University (Ethical Code: 20110530(2)). The study was registered with Chinese Clinical Trial Registry (ChiCTR): CTR 20132482.

### 2.2. Subjects and Study Design

Participants were recruited from Outpatient Department of Xiangya Hospital of Central South University in China between September 2011 and December 2012 via health promotion events, advertisements (local newspapers, notice boards, and homepages of the hospital), and telephone calls. The patients were diagnosed as FD according to RomeIII criteria. The Rome III diagnostic criteria for functional dyspepsia are shown in Table 1. The exclusion and inclusion criteria are listed in Table 2. Medical histories were acquired from all patients. Physical examination, gastroscopy, and laboratory tests were conducted. All participants signed the informed consent. Participation was free, and patients could withdraw at any time point when he/she feels unable to continue. Lifestyle advice was provided for the patients, and they were free to receive other health-care services from the center. Personal information of the patients was kept confidential before, during, and after the study.

### 2.3. Population

With a statistical power of 80% and a one-sided significance level at 5%, this study required a minimum recruitment sample size of 82 patients assuming 15% of the subjects would drop out or fail to follow-up [15].

### 2.4. Withdrawal, Discontinuation, and Dropout

The patients were free to withdraw from the trial, and the investigator can determine the time to terminate the trial when necessary. Explanations of the withdrawal were recorded in CRFs (case report forms), and the data collected before withdrawal were kept for analysis. The trial was terminated if one or more of the following conditions were met: (1) severe adverse events (AEs) of the drugs, (2) patients' request, (3) poor compliance to the medication, and (4) request for other drugs to alleviate FD symptoms.

### 2.5. Randomization and Blinding

All the participants were randomly assigned to four groups by block randomization. The randomized list was produced by an independent statistician using Microsoft Excel and a block randomization method according to the previous report [16]. The clinical investigator chose the treatment modalities for each patient based on the randomization number. The patients, statisticians, clinical investigators, and other staffs were all blinded to the study. The blinded procedures were confirmed by the authorized investigation agency. Emergency envelopes which contained the randomization code were given to the investigators, who were supervised to fully implement the blinded procedures until the trial ended. Grouping details were not uncovered until the trial ended unless in emergency.

### 2.6. Intervention

The results of previous clinical trials about drug safety and tolerance in human have shown that the maximum tolerance dose of single WKP administration to a healthy subject is 600 mg. The maximum tolerance dose of multiple WKP oral administrations to healthy subjects was 400 mg each time, 3 times daily. 100 mg flavonoid glycosides were contained in one WKP tablet. Besides, the effective dose of zhishi is 10 g or 15 g in clinical practice, which could be concentrated into 600 mg or 900 mg flavonoids and made into 6 or 9 tablets of WKP, respectively by Qingfeng Pharmaceutical Co., Ltd. (Jiangxi, China). Therefore, we determined that the subjects could take 2, 3, or 4 tablets of WKP, three times daily.

The patients randomly took either four tablets of WKP or placebo. The tablets were taken orally 30 minutes before three meals for 4 weeks.

  Placebo: 4 tablets of placebo, tid.
  Group 1–600 mg flavonoids: 2 tablets of WKP and 2 tablets of placebo, tid.
  Group 2–900 mg flavonoids: 3 tablets of WKP and 1 tablet of placebo, tid.
  Group 3–1200 mg flavonoids: 4 tablets of WKP, tid.

#### 2.6.1. Weikang Pian (WKP)

WKP tablets, prepared by the Qingfeng Pharmaceutical Company Ltd. (Jiangxi, China), were made from the flavonoids extracted from zhishi. 100 mg flavonoid glycosides were contained in one WKP tablet.

#### 2.6.2. Placebo

The placebo was provided by Qingfeng Pharmaceutical Company Ltd. (Jiangxi, China), which had the same dose, smell, taste, and appearance as WKP tablets but no active components. The patients were inquired about whether they knew the drug they took was WKP tablets or placebo at the end of the trial.

#### 2.6.3. Concomitant Medication

Drugs which may influence the outcomes of our study, including corticosteroids, GI motility-related drugs, and NSAIDs, were forbidden.

### 2.7. Follow-Up and Monitoring

Every 7 days, all the subjects were asked to return the remaining medicines and received the medicines to be taken. In this process, researchers emphasized that the medicines should be taken regularly. The symptoms of functional dyspepsia were assessed once a week. A clinical research associate (CRA) monitored and ensured data quality weekly, who reviewed the informed consents, medical records, electronic case report forms, and source documents.

### 2.8. Outcome Measures

#### 2.8.1. Primary Outcome Measures

FD symptoms were evaluated by the SDS scale (single dyspepsia symptom scale), which assessed 4 main FD symptoms (epigastric algesia, epigastric burning, early satiety, postprandial fullness, and bloating) from 3 aspects (intensity, discomfort, and frequency) with four levels (severe = 3, moderate = 2, mild = 1, and absent = 0). The sum of the three scores was defined as SDS scores. Both patients and the investigator rated at the baseline, the 1^st^, 2^nd^, 3^rd^, and 4^th^ weeks, respectively.

#### 2.8.2. Secondary Outcome Measures

The gastric emptying function (GEF) was considered as the secondary outcome. GER and GET/2 were evaluated with Radionuclide Imaging. Twenty barium markers and standard meals (45 g pork intestines and 97 g instant noodles) were given to the patients after fasting for 6–8 h. Abdominal radiographs were taken two hours later. The amount of markers remaining in the gastric cavity was counted, and gastric emptying function was estimated by observing the 2-hour gastric emptying rate and gastric semiempty time.

### 2.9. Safety and Compliance Assessment

The degree of subject compliance was assessed by “count pill” strategy. The ratio of the number of tablets that participants have actually taken to the number of tablets that should have taken was assessed, and the results were considered as the compliance to treatment.

To assess drug safety, blood biochemical examination (serum creatinine concentration (Scr), blood urea nitrogen (BUN), aspartate aminotransferase (AST), and alanine aminotransferase (ALT)), routine urine test, routine blood test, routine fecal test, and electrocardiogram (ECG) were performed before and after the treatment. Throughout the trial, adverse events (AEs) were carefully observed and recorded in CRFs. AEs were defined as any adverse and unexpected changes in symptoms, vital signs, or laboratory test results after participation in the trial. All patients were required to report any adverse events, which were recorded in case report forms. In addition, causes of all adverse events were evaluated. When severe AEs appeared, the principal investigator and the Institutional Review Board (IRB) would be informed immediately. And the investigator decided whether the patient was withdrawn from the research.

### 2.10. Fingerprinting Analysis

Three of the standard compound solutions (naringin, neohesperidin, and hesperidin) were prepared at seven concentrations diluted in methanol and stored at 4°C. The calibration curve was plotted via measuring the peak areas in seven concentrations of all reference compounds.

Quantitative analysis was performed using a Waters Acquity UPLC system (provided by Waters Corporation, Milford, America), with an automatic sample processing system (2695), Acquity BEH C18 column (1.7 *μ*m; internal diameter, 100 × 2.1 mm), Empower 2.0 data processing software, and a diode array detector (2996). The mobile-phase conditions contained methanol (A) and acetic acid (0.5%) in water (B). The gradient flow: 0-1 min, 0–30% A; 1–4 min, 30–50% A; and 4-5 min, 50–100% A. The analysis was conducted at a flow rate of 0.2 ml/min and detected at a wavelength of 284 nm. The temperature was kept at 4°C, and the room temperature was 25°C. The amount of each injection was 6 *μ*L.

### 2.11. Statistical Analysis

The intention-to-treat (ITT) analysis was performed on all participants who had taken at least one dose of the medication. Quantitative variables were described as mean ± standard deviation (SD). The independent sample *t*-test was used to analyze the efficacy between placebo and WKP groups. All statistical analyses were conducted with SPSS 13.0, and *P* < 0.05 indicated significant difference.

## 3. Results

### 3.1. Demographic Characteristics of the Patients

A total of 82 patients were recruited in this study, among which 22 were excluded because of various reasons. 60 patients were eventually enrolled in the trial and randomly assigned to four groups, placebo group (*n*=15, 7 males and 8 females), group 1 (*n*=15, 8 males and 7 females), group 2 (*n*=15, 6 males and 9 females), and group 3 (*n*=15, 5 males and 10 females). One patient in group 3 was withdrawn from the trial. The first patient entered the study in October 2011, and the last one entered in September 2012. Baseline characteristics of patients in the four groups are listed in Table 3. There was no significant difference between four groups in age, gender, characteristics, symptom scores, or course of disease before treatment.

### 3.2. Participant Flow

**Figure 1:**
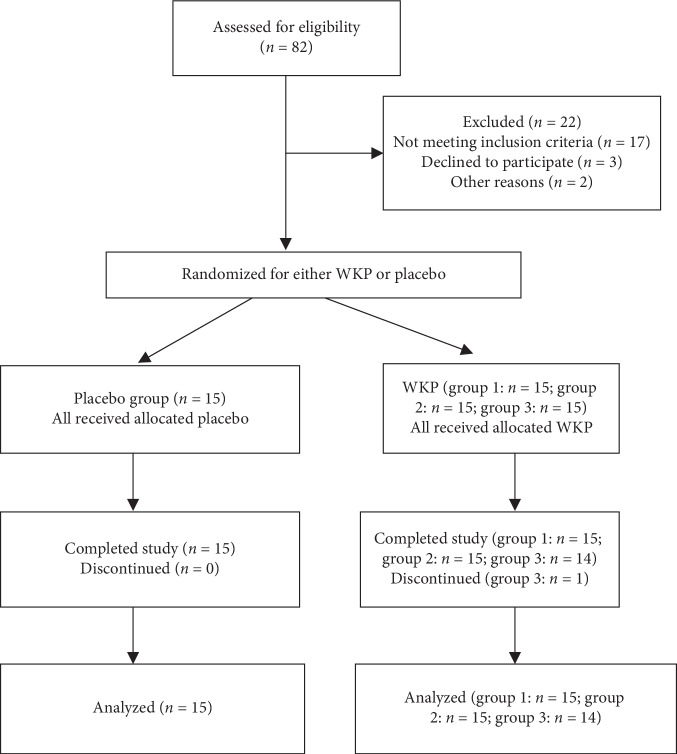
The flowchart of trial.

The flow of subjects in this study is summarized in Figure 1, and this trial followed CONSORT guidelines.

### 3.3. Primary Outcomes and Secondary Outcomes

**Figure 2:**
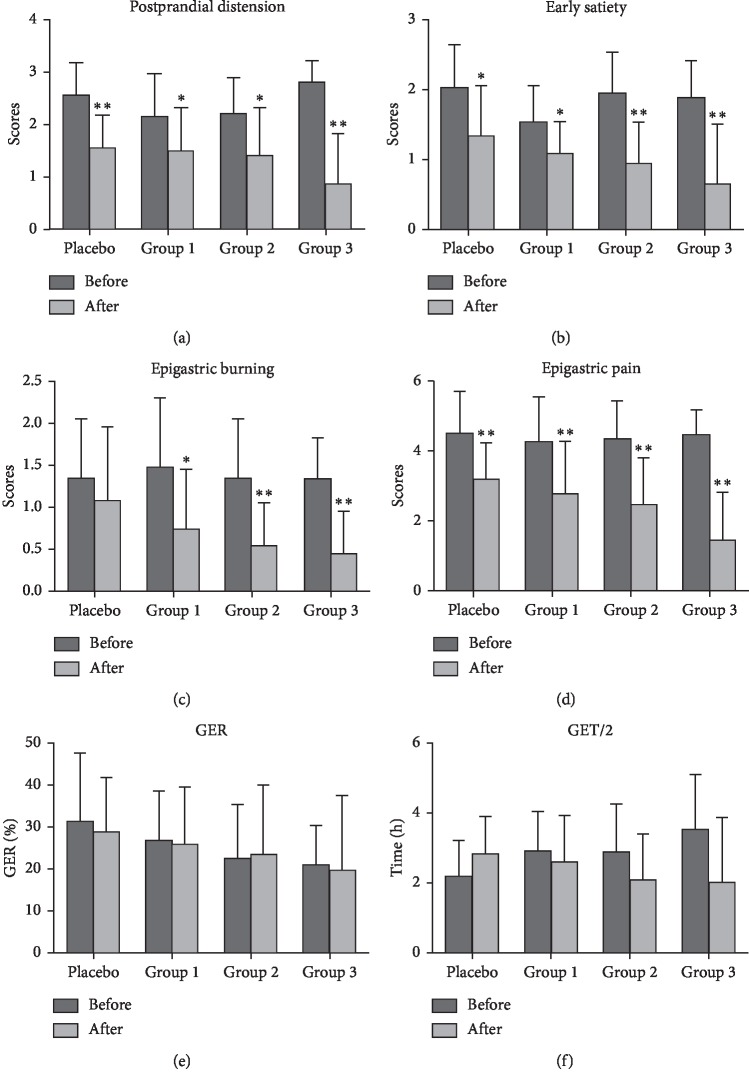
Changes of parameters: (a) postprandial distension; (b) early satiety; (c) epigastric burning; (d) epigastric pain; (e) gastric emptying rate (GER); (f) half gastric emptying (GET/2). ^*∗*^*P* < 0.05 vs. baseline respectively; ^*∗∗*^*P* < 0.01 vs. baseline respectively.

The primary outcome was SDS, and the secondary outcome was GEF. Dyspeptic symptoms were measured after the 4-week treatment. Evaluated symptoms include postprandial distension, early satiety, epigastric burning, and epigastric pain; scores decreased in all treatment groups from baseline to endpoint (Figure 2). Three symptoms containing postprandial distension, early satiety, and epigastric pain at the endpoint were significantly decreased in all treatment groups compared with the baseline (Figures 2(a), 2(b) and 2(d)). Epigastric burning was significantly alleviated in three WKP treatment groups, but no significant changes were found in placebo group (Figure 2(c)). There was no significant decrease of GER and GET/2 in the four treatment groups (Figures 2(e) and 2(f)).

**Figure 3:**
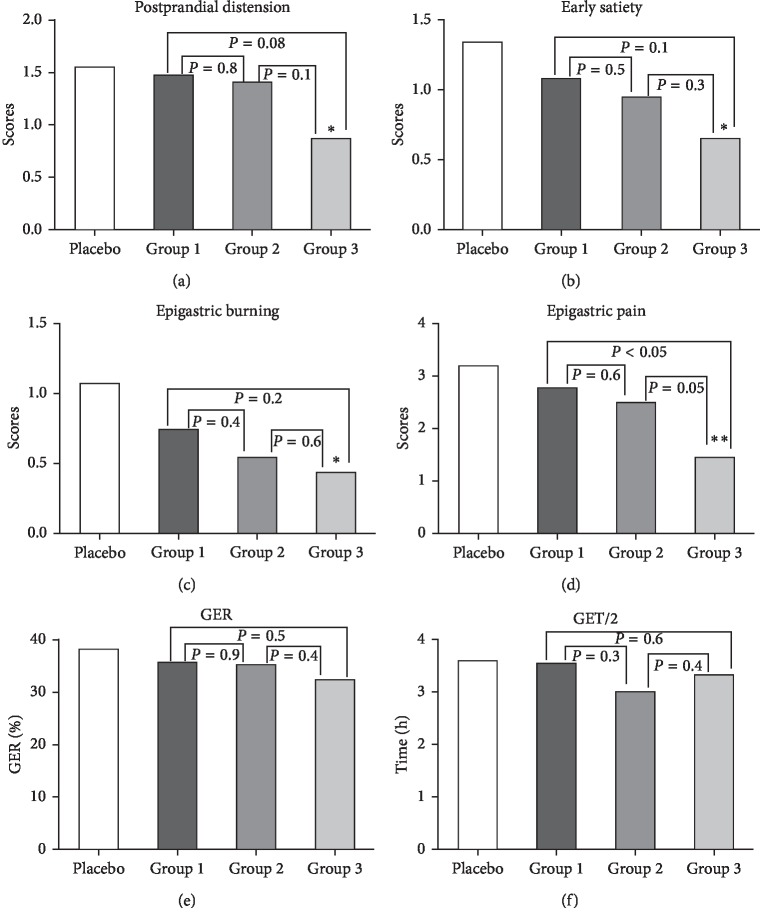
Scores at the endpoint: (a) postprandial distension; (b) early satiety; (c) epigastric burning; (d) epigastric pain; (e) gastric emptying rate (GER); (f) half gastric emptying (GET/2). ^*∗*^*P* < 0.05 vs. placebo; ^*∗∗*^*P* < 0.01 vs. placebo.

**Figure 4:**
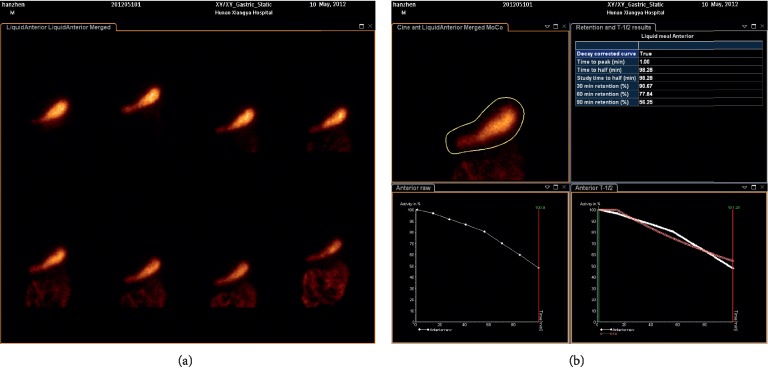
Radionuclide imaging of gastric emptying.

Scores of symptoms at the end point were compared among the four treatment groups. Compared with the placebo group, there were significant differences in symptoms of postprandial distension, early satiety, epigastric burning, and epigastric pain in group 3 but not in the other two WKP treatment groups. After 4 weeks of treatment, dyspepsia was relieved in a dose-dependent manner. The scores of symptoms decreased as the dose increased. There was no significant difference in four dyspeptic symptoms among the three WKP treatment groups. Besides, there was a significant difference in epigastric pain between group 1 and group 3 (Figures 3(a)–3(d)). In addition, there was no significant difference among the four treatment groups in gastric emptying parameters of GER and GET/2 (Figures 3(e) and 3(f)).  Figure 4 was an image of one patient.

### 3.4. Safety and Compliance

There were no study-related adverse events. Although one patient caught cold and another suffered from urinary tract infection, both were evaluated by the principal investigator and the Institutional Review Board (IRB). The events were considered to be irrelevant to the drug finally.

The compliance to treatment was 94%–96%. The compliance in placebo group, group 1, group 2, and group 3 was 95.24% (320/336), 94.05% (316/336), 94.94% (319/336), and 96.13% (323/336), respectively. There was no significant difference between the groups.

### 3.5. Phytochemical Analyses

**Figure 5:**
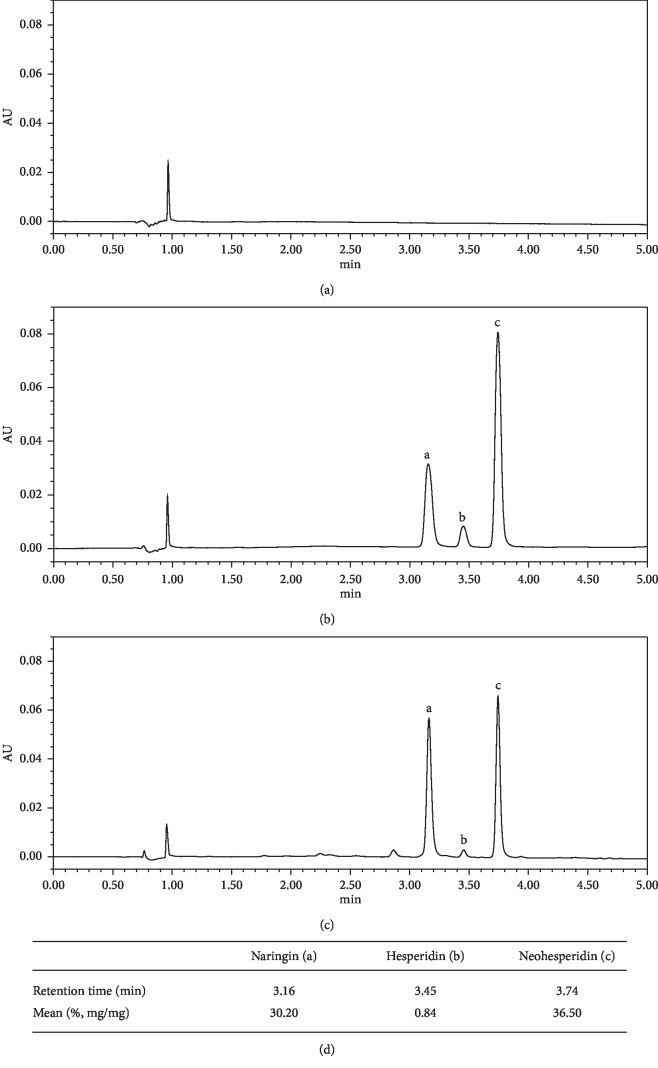
Fingerprint analysis of WKP with ultra-performance liquid chromatography (UPLC). (a) the methanol; (b) the standard solution; (c) WKP; (d) percentage composition.

Three kinds of flavonoid glycoside were analyzed by UPLC A (Figure 5). The percentage of naringin, hesperidin, and neohesperidin in WKP was 30.20%, 0.84%, and 36.50%, respectively.

## 4. Discussion

According to Rome III criteria, FD is defined as the symptoms in gastroduodenal regions (postprandial fullness, burning, early satiation, or epigastric algesia) exclusive of any metabolic, systemic, or organ diseases. Although FD is not life-threatening, life quality is dramatically affected. Pharmacotherapy includes simple and compound formulations, H2 blockers, prokinetics, proton pump inhibitors, mirtazapine, and antidepressants, which may relieve symptoms of certain patients [17, 18]. However, there are no satisfactory therapies at present. Herbal formulations to FD have been widely applied in China and some other countries.

Recently, certain antidepressants, e.g., mirtazapine, have been applied to FD treatment [19]. FD symptoms can be relieved by the formulation of Nigella sativa, ginger, artichoke leaf extracts, and honey [20, 21]. In recent years, the therapeutic advantages of TCM have been affirmed by more and more researches [22–24]. Studies have proved that Liu jun zi decoction, Jollab, and Xiangsha Liujunzi granules were effective in FD treatment; in addition, many reports have published application of CHM to FD treatment [17, 25–27].

In this study, we evaluated the efficacy and safety of WKP in patients with FD. A total of 60 FD patients were eventually enrolled in the trial, among them 45 patients were in the WKP group and 15 patients in the placebo group. The subjects randomly received WKP or placebo for 4 weeks with 4 tablets each time, 3 times daily. The primary outcome was the SDS scale, including assessments of postprandial distension, early satiety, epigastric burning, and pain. The secondary outcome was the gastric emptying function. Of note, the pathophysiological mechanism of FD remains unclear, and several mechanisms may be related to the disease, for instance, accommodation/emptying disorder of stomach, diet, gastric acid, infection, cytokines, genes, anxiety/depression, duodenal sensitivity, and cerebral modulating circuits of algesia [6]. The primary cause of FD in Asians is gastric motility disorder, such as antral dysmotility, delayed gastric emptying, and damaged meal accommodation [28]. It is reported that bloating is caused by excessive gas accumulation in the gastric cavity and intestine, while belching prevents gas accumulation under the physiological status [29]. Belching is frequently observed in FD patients, and the incidence reaches 80% [30, 31]. Compared with the healthy control group, FD patients swallowed more air, and more belches occurred [32], which might be a response to uncomfortable gastrointestinal sensations [33]. In our study, three symptoms containing postprandial distension, early satiety, and epigastric pain at the endpoint were significant decreased in all treatment groups compared with the baseline. Epigastric burning was significantly alleviated in the three WKP treatment groups, but no significant changes were found in the placebo group. In fact, it is difficult to assess outcomes of FD. The diagnostic criteria of FD, RomeIII criteria, is mostly based on patients' subjective symptoms, but lack of definitive laboratory diagnosis, which may result in subjective bias during the trail. Compared with the placebo group, there were significant differences in symptoms of postprandial distension, early satiety, epigastric burning, and epigastric pain in group 3. In the study about the FD, it was difficult to verify the effects of WKP on the overall FD symptoms, since there was a relatively high response rate in the placebo group. Generally, it has been reported that the placebo response rate reaches 30–40% in many FD clinical trials [34], which may be explained by some possible factors, such as natural history, smaller sample size, higher expectation, longer administration duration, too many visits, and harmonious therapeutic relationship [35]. In summary, WKP was effective in relieving FD symptoms, but the optimal dose could not be determined in our study since the sample size was not large enough. Moreover, there was no significant difference in GER and GET/2, but scores of GET/2 decreased in WKP treatment groups. However, a greater decrease in the scores was detected in patients treated with 1200 mg flavonoids per day.

As we know, WKP is made of flavonoids which are extracted from zhishi (Fructus Aurantii Immaturus). The gastrointestinal mucosa is the first barrier for digested xenobiotics and food. Much evidence have supported that natural phytochemicals, such as flavonoids, have bioactivity and potential health benefits, e.g., antioxidant, antibacterial, and anti-inflammatory effects, thereby preventing digestive diseases. Resveratrol (3,5,4′-trihydroxystilbene) is a member of flavonoids that could alleviate the gastric mucosa induced by ischemia/reperfusion by increasing Ca^2+^-ATPase and Na^+^-K^+^-ATPase [36]. In addition, iNOS, NF-*κ*B, and IL-8 expressions enhanced by *H. pylori* (108 CFU) were inhibited by resveratrol via activating Nrf2/HO-1 pathway in the gastric of mice [37]. Pogostone is another type of flavonoid and has an impact on gastrointestinal motility, whose mechanism is possibly related to the enhanced cellular antioxidant activity and PGE2. Pogostone may increase gastric GSH, catalase, and SOD levels and decrease LPO and mucosal apoptosis [38]. It has been shown that Aurantii fructus immaturus flavonoid (AFIF) could improve the contraction of isolated gastric smooth muscle strips in rats, which has a diastolic effect on PCSMS. This effect is closely related to the NOS activation, cGMP and PKG upregulation, and decrease of intracellular Ca^2+^ concentrations in smooth muscle [39].

The concentrations of naringin, hesperidin, and neohesperidin in WKP are 30.20%, 0.84%, and 36.50%, respectively. In one study, naringin contained gastroprotective properties and involved in repair of mucosal injury induced by absolute ethanol [40]. It was recently reported that the neohesperidin and hesperidin had potential anti-inflammatory effects and may mitigate gastric injuries elicited by indomethacin [41].

Theoretically, FD symptoms may be relieved via the mechanisms below: modulating central or peripheral algesia pathways, reducing dysmotility, and relaxing smooth muscle which leads to enhanced gastric accommodation and gastric fundus relaxation. Since oxidative stress and inflammation may be related to the pathogenesis of FD [6, 42], the antioxidant and anti-inflammatory properties of WKP might explain the antidyspeptic effect. FD is associated with *H. pylori* infection, but very few patients (1/17) are relieved following *H. pylori* eradication [6]. Thus, *H. pylori* eradication might also partially explain WKP's effect.

There are some limitations in our study. For instance, the effectiveness and safety of WKP have been evaluated; however, the sample size was too small to evaluate the optimal dose, and the duration of intervention was not long enough to observe whether there was significant difference between the WKP and placebo groups.

## 5. Conclusion

WKP was effective in relieving FD symptoms, but the optimal dose could not be determined in our study since the sample size was not large enough. Additional data and further study are required to verify our findings. It remains to further explore about the optimal dose and the precise mechanisms of WKP in FD treatment.

## Figures and Tables

**Table 1:** Rome III diagnostic criteria for functional dyspepsia.

Functional dyspepsia	Subtype
The last 3 mo with symptom onset at least 6 mo before diagnosis, and must include: (1) One or more of: (a) Bothersome postprandial fullness (b) Early satiation (c) Epigastric pain (d) Epigastric burning AND (2) No evidence of structural disease (including at upper)	Postprandial distress syndrome Must include one or both of the following: (1) Bothersome postprandial fullness, occurring after ordinary sized meals, at least several times per week (2) Early satiation that prevents fishing a regular meal, at least several times per week Supportive criteria (1) Upper abdominal bloating or postprandial nausea or excessive belching can be present (2) EPS may coexist

**Table 2:** Inclusion and exclusion criteria.

*Inclusion criteria*	(i) Aged 18–65 years, either sex
	(ii) Agreed with study and signed informed consent
	(iii) Clinical diagnosis of FD, according to functional dyspepsia criteria
	(iv) Stopped administrating prokinetic drugs, gastric mucosa protectant and hydrotalcite in the past 28 days

*Exclusion criteria*	(i) Patients with oesophagitis, atrophic gastritis, gastric and duodenum ulcers
	(ii) Patients with liver, gallbladder, pancreas organic diseases
	(iii) Patients with diabetes, kidney diseases, connective tissue diseases and psychosis
	(iv) Patients with heart, brain, lung, kidney, hemopoietic system and endocrine system primary diseases
	(v) Patients with abdominal operation
	(vi) Patients with renal insufficiency, score of creatinine more than upper limit of normal
	(vii) Patients with hypohepatia, score of alanine aminotransferase ≥1.5 × upper limit of normal
	(viii) Current pregnancy or lactation
	(ix) Psychopaths and disability in law
	(x) Patients participated in another clinic trial in past 3 months

**Table 3:** Baseline of characteristics.

Parameters	Placebo	Group 1	Group 2	Group 3
Gender (male/female)	7/8	8/7	6/9	5/10
Age (year)	40.40 ± 8.09	38.67 ± 9.29	39.07 ± 7.85	38.60 ± 9.34
Weight (kg)	60.73 ± 6.80	60.99 ± 7.49	59.29 ± 6.20	59.68 ± 6.71
Height (cm)	165.07 ± 4.85	165.33 ± 6.99	162.20 ± 5.82	163.87 ± 6.08
Smoking (yes/no)	4/11	4/11	3/12	2/13
Postprandial distension	2.53 ± 0.64	2.13 ± 0.83	2.20 ± 0.68	2.80 ± 0.41
Early satiety	2.00 ± 0.65	1.53 ± 0.52	1.93 ± 0.59	1.87 ± 0.52
Epigastric burning	1.33 ± 0.72	1.47 ± 0.83	1.33 ± 0.72	1.33 ± 0.49
Epigastric pain	4.49 ± 1.21	4.25 ± 1.28	4.33 ± 1.08	4.47 ± 0.69
GER (%)	42.62 ± 19.47	35.01 ± 18.57	31.41 ± 13.08	27.41 ± 14.01
GET/2 (h)	2.89 ± 1.39	3.70 ± 2.04	3.85 ± 1.92	4.63 ± 2.36

Group 1-2 tablets of WKP, 600 mg flavonoids; Group 2-3 tablets of WKP, 900 mg flavonoids; Group 3-3 tablets of WKP, 1200 mg flavonoids.

## Data Availability

The datasets used and/or analyzed during the current study are available from the corresponding author on a reasonable request.

## Abbreviations

WKP: Weikang pian
FD: Functional dyspepsia
CHM: Chinese herbal medicine
CRFs: Case report forms
AEs: Severe adverse events
CRA: Clinical research associate
SDS scale: Single dyspepsia symptom scale
GEF: The gastric emptying function
UPLC: Ultraperformance liquid chromatography
ITT: The intention-to-treat.
